# Observations on Yaws, and Its Influence in Originating Leprosy; Also Observations on Acute Traumatic Tetanus, and Tetanus Infantum

**Published:** 1840-04

**Authors:** 


Art. II.—*Observations on Yaws, and its Influence in Originating Leprosy; also Observations on Acute Traumatic Tetanus, and Tetanus Infantum.* By James Maxwell, m.d.—*Edinburgh*, 1839. 8vo, pp. 134.

This treatise on yaws was an inaugural dissertation, for which the senators of the university of Edinburgh awarded their gold medal to the author. Judging from the internal evidence afforded by the work itself, it seems to be written by a practitioner of considerable experience, not as these productions usually are, the compilation of a mere student. It is the most complete treatise which has yet appeared on a formidable disease which in this country has attracted no attention, as fortunately it is unknown; and even where it is prevalent, has been much disregarded by British surgeons, and left to the care of the blacks themselves. Those who are liable to be called on to treat the disease, would do well to read Dr. Maxwell's book, and to try his remedy, hydriodate of potash. Appended is a sensible paper on tetanus infantum, which disease, from its extreme frequency and fatality, has always been one of the chief causes of decrease among the West Indian negroes. Dr. Maxwell found, from many years' observations, that twenty-five per cent. of all the negro children died of this disease in Jamaica. Dr. Hancock has asserted that it kills half the infants in many of the other islands. According to Dr. Maxwell, the sole cause is the neglected state of the navel, and that the disease may be prevented. The midwives apply turpentine, tobacco ashes, bark, &c., to the funis, wrap it up in burnt rag, apply a bandage, and give strict orders not to remove the dressing until the funis drops. With admirable consistency, they give the infant rum and castor oil until the ninth day, and keep it and the mother in a room from which air is excluded, and then light a fire.

The following extract illustrates Dr. Maxwell's prophylactic treatment, and the success with which it was followed :

"I was consulted by a lady, on account of the ravages which the disease was making on her plantation; she informed me that twenty-five infants had perished in a few years from locked-jaw. The women were removed from the negro-houses, and delivered in one of the airy apartments of the mansion-house, under her immediate superintendence; yet, notwithstanding all her care and attention, the mortality was as great as it had been under less favorable circumstances. The treatment was, to give the child a dose of castor oil shortly after birth, to dress the navel with bark, and each day to add a fresh portion to absorb the acrid matter, without washing it till it dropped. I advised her to change her method of dressing the funis, and to bathe it carefully with milk and warm water once a day, or twice if it was large, and after each ablution to apply a pledget of cerate or spermaceti ointment, carefully abstaining from purgatives and irritant dressings. Slight as this alteration was, she entered upon it with a determination to give it a fair trial, and her efforts were crowned with perfect success; for she lately informed me that, since its adoption, for several years past, she has never lost a child from tetanus."

There are few curses, we are daily convinced, more dreadful than the curse of nurses. The elderly ones are the incarnation of worn-out prejudice; every absurdity of a bye-gone day is treasured up and transmitted by some of these people, who like filters catch all that is worthless, and let the clear liquor run through. Some centuries ago the worst of them were called witches, and were burnt, and there was "wild justice" certainly in such treatment. Slavery is bad enough, but what are the whips and scorns of slave-drivers to grown-up men, compared to this turpentine to the tender navels of sensitive babies, with rum and castor oil for food, and a close black-hole warmth, ending in tetanic convulsions, and a mortality of twenty-five per cent.? The sufferings of white infants are not few or inconsiderable from the same sources : what with castor oil, opiates, gruel, gin and sugar, bandages round their chests to "support" them as they call it, and such like means, having all the authority of tradition; but these sink into nothingness, when compared with the deeds of the black midwives. If Dr. Maxwell has at all succeeded in introducing, even partially, a better system, he will have overcome no slight difficulties, and is deserving of much praise.

